# An Advanced Molecular Detection Roadmap for Nonlaboratorians

**DOI:** 10.3201/eid3113.241506

**Published:** 2025-05

**Authors:** Jessica N. Ricaldi, J. Todd Parker, Nathelia Barnes, Hannah Turner, Scott Santibañez

**Affiliations:** Centers for Disease Control and Prevention, Atlanta, Georgia, USA

**Keywords:** Advanced molecular detection, public health, infectious disease

## Abstract

This article, aimed at nonlaboratorians such as healthcare providers, public health professionals, and policymakers, provides basic concepts and terminology to enable better understanding of other manuscripts in this advanced molecular detection journal supplement. This article focuses on 3 aspects of advanced molecular detection: pathogen genomics, bioinformatics, and public health application, while providing additional resources for understanding.

Advanced molecular detection (AMD) combines next-generation sequencing (NGS), bioinformatics, and traditional epidemiology to provide detailed information on disease-causing microorganisms, or pathogens (*1*). AMD has become central to the US public health system’s efforts to identify, track, and stop infectious diseases. The Centers for Disease Control and Prevention’s (CDC) Office of Advanced Molecular Detection, part of the Division of Infectious Disease Readiness and Innovation, National Center for Emerging and Zoonotic Infectious Diseases, works to modernize the public health system’s disease investigation capabilities by using the latest technologies and building AMD capacity in public health partner institutions (*1*).

Although AMD has empowered public health agencies across the United States to rapidly identify and solve outbreaks that were previously undetectable, the technical terminology can be challenging for many healthcare and public health professionals. This article aims to provide nonlaboratorians such as physicians, advanced practice providers, public health professionals, and policy makers with an overview of how advanced molecular approaches are used to detect and control infectious disease threats. This primer will assist nonlaboratory personnel to better understand the concepts and terminology used in this AMD journal supplement, as well as in the daily practice of clinical medicine and public health.

We will focus on 3 aspects of AMD: pathogen genomics, how laboratory scientists use technologies to study the genetic composition, or sequences, of infectious microorganisms; bioinformatics, how high-performance computing is used to analyze genetic sequence data; and public health application, how epidemiologists, clinicians, and other public health professionals combine information from field investigations with genetic sequence data to identify and stop outbreaks. Although much has been written about the use of NGS for mapping the human genome, the focus of this journal supplement is pathogen genomics, the sequencing of microorganism genomes that can cause infectious diseases.

## Three Aspects of AMD

### Pathogen Genomics

As recently as the late 20th Century, healthcare providers and clinical laboratories relied on established, culture-dependent techniques for the laboratory identification of bacteria and viruses and the reporting of such findings for disease surveillance. Sanger sequencing is a method for DNA sequencing of specific genes developed in the 1970s. Sanger sequencing is highly accurate but expensive and time-consuming, especially when sequencing an organism’s entire genetic code or genome (*2*). The development of NGS in the early 2000s greatly advanced the field of genome sequencing and analysis, or genomics. NGS enabled the rapid, automated sequencing of many genetic fragments in parallel, providing a large amount of genetic information rapidly and at a lower cost compared with older methods. A wide range of approaches to sequencing have since been developed that can be targeted to look for a specific pathogen or be pathogen agnostic and sequence any microbial genetic material in a sample. Some examples of commercially available laboratory sequencing methods include detection of fluorescently labeled nucleotides (Illumina, https://www.illumina.com), detection of hydrogen ions during polymerization (Ion Torrent, https://www.thermofisher.com), analysis of electrical signals from biologic molecules that have passed through nanometer-sized pores (Oxford Nanopore, https://www.nanoporetech.com), and direct observation of the sequencing process (PacBio, https://www.pacb.com). To date, available sequencing systems, or platforms, can be broadly grouped into short-read or long-read platforms on the basis of the length of sequence reads they produce, measured in base pairs. A base pair is a unit of double-stranded nucleic acids consisting of 2 complementary DNA nucleotide bases bound to each other by hydrogen bonds. Short-read platforms (<500 bp) fragment the genome to be sequenced into short fragments and are more reliable for detecting low frequency genetic variations and short insertions, deletions, and mutations (*3*). Long-read methods (3,500–11,000 bp) can read longer stretches of DNA, or complete regions of a gene, and are used when studying complex genomes such as in metagenomic sequencing, which involves analysis of genetic material of all organisms that may be within environmental or clinical samples. Short-read platforms are better for identifying the precise genome sequences, nucleotide by nucleotide, whereas long-read platforms are better for identifying large DNA insertions or deletions (*4*).

NGS involves both traditional laboratory components, such as sample collection, DNA extraction, and sequencing machines, and bioinformatics components, such as using computational models, also known as pipelines, to analyze the large volumes of data created by NGS. Analysis of these data can reveal epidemiologic patterns of disease transmission, genetic variations, antimicrobial resistance genes, and other information necessary to clinical care and public health. As NGS technologies became more widely available and affordable, sequencing whole bacterial and viral genomes to understand disease transmission became common in clinical and public health laboratories (*2*). Along with increased use, validation of NGS tests is both critical and difficult because of workflow variations across laboratories, such as differing sample types; operating procedures for extraction, amplification, and sequencing; and bioinformatic processes. Because of those differences, specific quality parameters are vital for both laboratory sequencing and bioinformatic technologies. CDC has invested in the development of quality management systems and quality system tools that are both technology and manufacturer specific.

Whole-genome sequencing (WGS), a type of NGS, enables scientists to determine a mostly complete sequence of an organism’s genome and provides more data than methods that only sequence a portion of the genome. For example, in addition to providing information about the evolutionary history and relationships among streptococcal organisms, potential streptococcal drug resistance patterns and typing (e.g., M protein typing) can be genetically inferred by using the same WGS pipeline (*5*). WGS has also improved surveillance for foodborne pathogen outbreaks and enhanced the detection of trends in foodborne infections and antimicrobial resistance at the state public health laboratory level.

An additional application that has moved from research to clinical practice is 16S sequencing. The 16S ribosomal RNA gene is conserved and found in all bacteria and is the most widely used for phylogenetic identity of a bacteria and the most frequently ordered of advanced molecular tests (*6*). Scientists amplify, sequence, and compare it with other known 16S sequences, using 16S variable and conserved regions for clinical laboratory diagnosis. Whereas WGS is also used for viral sequencing, ribosomal RNA sequencing enables many bacteria to be identified at the genus or species level, including bacteria that are hard to cultivate or following the administration of antimicrobial therapy (*7*).

## Bioinformatics

NGS provides a large amount of genetic information. Microbial bioinformatics is a data-driven approach that combines the use of sequencing data, machine learning, and artificial intelligence for rapid public health response. This field uses computational tools for disease surveillance, monitoring antimicrobial resistance, and outbreak investigations. By using computer science and statistical methods, such as high-performance supercomputing to organize and interpret the data, bioinformatic tools can track, identify, and monitor pathogens while tracing transmission pathways and phylogenetic origins. Phylogenetic methods play a crucial role in studying the evolutionary history and relationships among organisms. Bioinformatic pipelines are used to assemble genomes, detect genetic variants, and build phylogenies, which are visual representations of the evolutionary relationships among organisms. Those pipelines start with a defined set of files, such as FASTA sequences (a text-based format that represents nucleotide sequences). Connected software routines are then used to generate results, such as sequence alignment or tree figures. Different tools and workflows can also be used to assemble a genome or to perform variant calling (i.e., detecting variants by comparing against a reference genome) (*8*).

Alignments can identify genetic variations such as single-nucleotide polymorphisms, which are variations in a single nucleotide at a specific place on the genome. Alignments of specific gene sequences or whole genomes aligned to a reference sequence are used as the input for the software or pipelines that generate phylogenies, which trace patterns of shared ancestry among organisms. By analyzing phylogenies, researchers can infer relatedness between pathogen sequences and describe them by using graphics and diagrams such as phylogenetic trees, which illustrate the genetic relationships among organisms. Phylogenetic trees are built by using different probability methods for analysis with various software developed to ensure computational efficiency (*8*). Phylogenetic trees that show relatedness among pathogens from different sources can provide additional information to complement traditional epidemiology data, determine associations, and help link human cases or establish a common source of infection.

Commercially available bioinformatics pipelines are often used for clinical diagnostic testing. Such pipelines must comply with patient safety, laboratory quality assurance, comparability across laboratories, and local and federal regulatory compliance requirements (*9*). Many open-source tools, such as Nextstrain (https://www.nextstrain.org), UShER (https://www.genome.ucsc.edu/cgi-bin/hgPhyloPlace), and MicrobeTrace (https://www. microbetrace.cdc.gov), and laboratory-developed code are also available and frequently used. Software containerization methods are used to package bioinformatics tools and pipelines into portable units (or containers), improving efficiency, reproducibility, and security (*10*), and to combine pathogen genomic information with other sources of information to estimate cases and predict the pathogen’s origins, movements, and potential affect (*11*).

Scientists have a critical need to share information quickly and efficiently. As a part of the National Institutes of Health, the National Center for Biotechnology Information (NCBI) serves a key role by providing access to biomedical and genomic information, as well as developing software tools for bioinformatics and sequencing analysis. NCBI has served as a hub for sharing genomic information through the GenBank DNA sequence database. The availability of sequences through GenBank enables scientists to compare sequences from other laboratories. AMD is now used to track a wide array of disease agents, including antimicrobial-resistant foodborne bacterial and fungal pathogens, including many in the NCBI Pathogen Detection isolate browser (https://www.ncbi.nlm.nih.gov/pathogens). Other examples of resources that enable sharing of information include the Virus Pathogen Database and Analysis Resource (https://www.bv-brc.org), a platform which provides information about virus mutation, and GISAID (https://www.gisaid.org), an online platform that enables the sharing of information about viral genomic sequences. Those resources play a crucial role in monitoring viral pathogens, including novel strains and respiratory viruses, contributing to the understanding of their evolution and transmission patterns.

## Public Health Application

Because sequencing costs decreased and platforms were created to manage and analyze larger sets of data, the use of those methods went from proof-of-concept and validating results against traditional epidemiology methods to becoming standard methodologies (*12*). AMD has become a central part of public health efforts to identify and control infectious diseases and is now incorporated into public health outbreak and emergency response, disease surveillance, drug resistance detection, clinical microbiology, and other public health applications (*13*).

In the United States, funding provided through CDC has been instrumental in building national capacity for AMD in state, local, and territorial public health laboratories, as well as in hospital and clinical laboratories. The use of AMD has evolved to include a wide array of infectious diseases, including respiratory diseases and antimicrobial drug resistant diseases (*14*,*15*). AMD has enhanced public health professionals’ ability to rapidly identify pathogens across the country, track the spread and identify sources of outbreaks, detect drug resistance in US hospitals, inform vaccine development, conduct disease surveillance, and promote international collaborations. Other reports have provided examples of linkages of AMD to surveillance and epidemiology in traveler-based genomic surveillance, enhanced AMR surveillance, and outbreak investigation (*16*–*18*). The global relevance of AMD and presence of similar programs in other countries is also of note. For example, a report on the national genomic surveillance system for *Listeria monocytogenes* and the effect of implementing decentralized sequencing in Australia is included in this issue (*19*). 

The following are a few examples of how AMD is used in the field of public health. First, PulseNet is a national laboratory network that uses AMD diagnostics to detect and prevent foodborne outbreaks (*20*). PulseNet International involves implementation of whole WGS for global food-borne disease surveillance (*21*). Second, the MinION portable DNA sequencer was used during the 2014 Ebola virus outbreak in West Africa (*22*). Third, the Secure HIV TRAnsmission Cluster Engine has been used to identify clusters of highly similar HIV sequences, indicating rapid transmission (*23*). Fourth, AMD-supported diagnostics were used in the early detection of SARS-CoV-2 variants (*24*). Finally, rapid AMD-supported diagnostic testing was used during the mpox outbreak response (*25*). 

A necessary part of applied public health is being aware of the caveats and limitations of methods and their applications, as well as understanding questions that public health practitioners should be asking when presented with data derived from NGS methods. Public health practitioners should consider several points when they are given data derived from NGS methods. First, practitioners should ask about the methods used when data are presented and be aware of the limitations of each. Each sequencing methodology has its own limitations. For example, if a lab provides sequencing data using nanopore, there are challenges in using those data for the detection of mutations. Second, practitioners should know a gene being detected doesn’t indicate the gene is being expressed or is functional. Third, practitioners must realize different methods will yield different results; for example, genome assemblies of the same organism by 2 different methods may provide different single-nucleotide polymorphism counts when run on the same panel and could affect epidemiologic investigations, and different bacterial or viral classifiers will yield different results from the same metagenomic raw data. Finally, practitioners should be aware that increasing use of metagenomics presents many unique challenges with results interpretation. When you receive results of interest consider the following: what the method or pipeline was built to do versus what is it doing (i.e., using a SARS-CoV-2 method to look for bacterial DNA); what the result is based on, a single gene or part of a gene; and what databases were used for the analysis. Many pathogens of interest are overrepresented in databases and may turn up disproportionally in results.

## Workforce Development and Capacity Building

Laboratory methods have evolved from relatively simple, culture-dependent techniques for identifying bacteria and viruses to expensive and time-consuming DNA sequencing to more rapid and less costly sequencing, resulting in vast amounts of genetic information. Information about those advances has not always been communicated clearly to healthcare and public health professionals or the public. For readers who are interested in learning more, CDC has enabled state, local, and territorial public health laboratories to provide regional training, including sequencing and molecular epidemiology tools trainings, Bioinformatics Regional Resources, and other opportunities designed to help clinicians, scientists, and public health practitioners to understand transmission chains, characterize emerging pathogens, and solve outbreaks (*1*) (Table).

**Table:** Education resources to further understanding of advanced molecular detection for nonlaboratorians

Resource title	Description	Link
Advanced Molecular Detection COVID-19 Genomic Epidemiology Toolkit	Toolkit to address topics related to the application of genomics to epidemiologic investigations and public health response to SARS-CoV-2.	COVID-19 Genomic Epidemiology Toolkit, https://www.cdc.gov/advanced-molecular-detection/php/training
American Society for Microbiology Comprehensive Training in Infectious Disease Applications of Next Generation Sequencing	Free training to educate the clinical microbiology workforce on next generation sequencing technologies to increase pathogen genomic sequencing capacity and increase preparedness for the next pandemic through enhanced molecular surveillance.	Training in NGS for Infectious Disease Applications, https://asm.org/Webinars/training-ngs-infectious-diseases
Association of Public Health Laboratories Advanced Molecular Detection	Resources for building the advanced molecular detection workforce and other related activities.	Advanced Molecular Detection, https://www.aphl.org/programs/infectious_disease/Pages/Advanced-Molecular-Detection.aspx
Association of Public Health Laboratories Learning Center	Multiple online courses on molecular testing and sequencing. Some courses are only available for members.	Learning Center, https://learn.aphl.org/learn/signin
Council of State and Territorial Epidemiologists Advanced Molecular Detection Workgroup	Information about public health professionals interested in using the latest next-generation genomic sequencing technologies at local, state, tribal, and territorial health departments for disease detection, surveillance, outbreak investigation and prevention. The workgroup includes a listserv.	Advanced Molecular Detection Subcommittee, https://www.cste.org/page/AdvancedMolecularDetection
Council of State and Territorial Epidemiologists Webinar Library	Multiple webinars developed by council of state and territorial epidemiologists.	Webinar Library, https://www.cste.org/page/WebinarLibrary
Northwest Pathogen Genomics Center of Excellence	Website includes publications, situation reports, tools, workflows, and other educational resources.	Northwest Pathogen Genomics Center of Excellence, https://nwpage.org
Minnesota Pathogen Genomics Center of Excellence	Website describes projects completed by the Minnesota Pathogen Genomics Center of Excellence.	Infectious Disease Projects, https://www.health.state.mn.us/diseases/idlab/pathogen
Virginia Pathogen Genomics Center of Excellence	Videos and other teaching resources.	Virginia Pathogen Genomics Center of Excellence, https://va-pgcoe.org
State Public Health Bioinformatics Group	Resources and materials from previous trainings offered.	StaPH-B, https://staphb.org

Beyond regional trainings, examples of other resources include CDC’s AMD Academy, hosted in partnership with the Association of Public Health Laboratories and the Council of State and Territorial Epidemiologists. The AMD Academy is a multiday training in molecular epidemiology and bioinformatics for epidemiologists and microbiologists from state, territorial, and local health departments. The COVID Genomic Epidemiology toolkit is another valuable resource that addresses topics related to the application of genomics to epidemiologic investigations and public health response to SARS-CoV-2 at state, territorial, and local levels. This toolkit provides introductory information such as how to interpret phylogenetic trees in the context of transmission, along with practical case studies demonstrating real-world applications (*26*). 

In conclusion, this journal supplement aims to improve knowledge and awareness of advances in AMD. The progress of AMD techniques over the past few decades has had a considerable effect on public health. We hope that this article can begin to demystify the field of AMD by providing a brief introduction to other papers in this journal supplement and AMD in general. 
